# What can GP data tell us about the treatment of onychomycosis in the UK?

**DOI:** 10.1002/ski2.84

**Published:** 2021-12-16

**Authors:** M. Sajeed, L. Wei, S. Murdan

**Affiliations:** ^1^ UCL School of Pharmacy University College London London UK

## Abstract

**Background:**

Treatment of onychomycosis is challenging, and there is much literature on optimal treatment strategies. In contrast, information on how onychomycosis is actually treated in primary care is scarce. Information on practice is important as it can reveal much, such as, to what extent national guidelines are followed and which population groups seek/receive treatment or do not do so.

**Objectives:**

To describe the pattern of onychomycosis treatment in primary care in the UK, by patient's gender and age.

**Methods:**

A population‐based retrospective cross‐sectional study was conducted. The Health Improvement Network (THIN) database was used to calculate incidence rates of onychomycosis in the years 2001–2017. The prescription of oral and topical anti‐fungal drugs to patients with onychomycosis was reviewed.

**Results:**

THIN data showed an onychomycosis incidence rate of about 50 per 100,000. More males than females (52% vs. 48%), and more people aged 50–59 years had received treatment for onychomycosis. Oral terbinafine was the most commonly prescribed drug, followed by topical amorolfine, although terbinafine was used more commonly by men and amorolfine by women. Patients with onychomycosis were also prescribed other antifungals, including itraconazole, griseofulvin, tioconazole, ketoconazole shampoo, fluconazole and clotrimazole. A greater proportion of women, compared to men, were prescribed fluconazole.

**Conclusions:**

Onychomycosis treatment in primary care in the UK is broadly in concordance with national guidelines.

## Graphical abstract

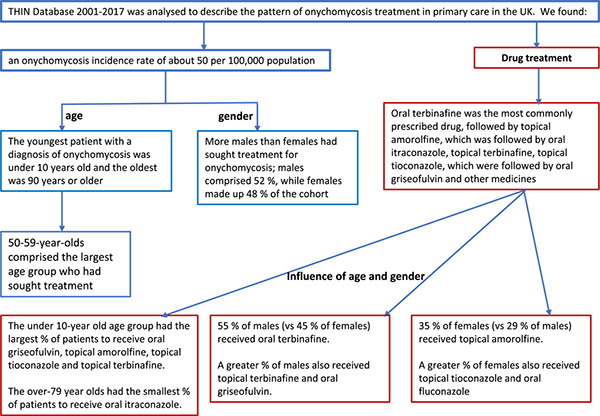

1

**What is already known about this topic?**

An attempt to offer global perspectives on the management of onychomycosis was conducted via a survey of expert physicians from Canada, Italy, UK, Israel, India, Brazil, and USA. This survey showed that the majority of experts used systemic, topical and combination treatments approved in their countries, based on product insert or government recommendations. In addition, although the basics of onychomycosis management was similar among the countries, slight differences existed related to adjunctive therapy, rating of disease severity and use of prophylaxis treatment (Gupta et al. 2019^14^).

**What does this study add?**

This study is specific to the UK and to onychomycosis treatment by General Practitioners. We show that GPs treat onychomycosis broadly in concordance with national guidelines. We also show that some patients with onychomycosis use anti‐fungal medicines that are likely prescribed for infections other than onychomycosis, which indicates the possibility of fungal infection spreading from one site to another.

## 2 INTRODUCTION

Onychomycosis, that is fungal infection of the nail, is a growing problem due to the global increase in populations with risk factors, such as old age, diabetes, immunocompromised status, as well as lifestyle factors, such as use of communal facilities such as gyms and swimming pools. Population‐based investigations have shown a wide range of onychomycosis prevalence, from less than 0.5% in Malawi and Zaire,^1^, ^2^ 2.7% in UK,^3^ 8.4% in Finland^4^ and a mean of 4.3% in Europe and North America.^5^ Onychomycosis can affect all age groups, but is more common in older adults, in males compared to females, and in toenails compared to fingernails.^5^ The dermatophyte *Trichophyton rubrum* and the yeast *Candida albicans* are the most common causative organisms of toenail and fingernail infections respectively, although the aetiology does differ geographically.^6^

Infected nails affect sufferers' quality of life; for example, they are a source of pathogens, complicate foot conditions in people living with diabetes, make walking and wearing footwear difficult, and unsightly fingernails cause emotional embarrassment.^7^, ^8^ Their treatment is however extremely challenging. The condition is chronic and recurrence (relapse following treatment and reinfection) is common, leading to onychomycosis being called stubborn,^9^ and much research on ungual drug delivery and the development of more effective medicines, for example Rizi et al. (2018).^10^ Oral antifungals are the mainstay of therapy with success rates of about 60%–70%,^11^ followed by topical antifungals for mild and superficial infections and when oral antifungals are contra‐indicated. Oral and topical medicines may be used in combination, which enhances success rates.^12^ Physical modalities including lasers and photodynamic therapies are also being investigated as alternatives.^13^ A global survey of dermatologists showed that the basics of onychomycosis treatment are similar around the world, with slight differences due to different approaches to adjunctive therapy, rating of the severity of disesase, use of prophylaxis treatment and regulatory approval of medicines.^14^

**FIGURE 1:**
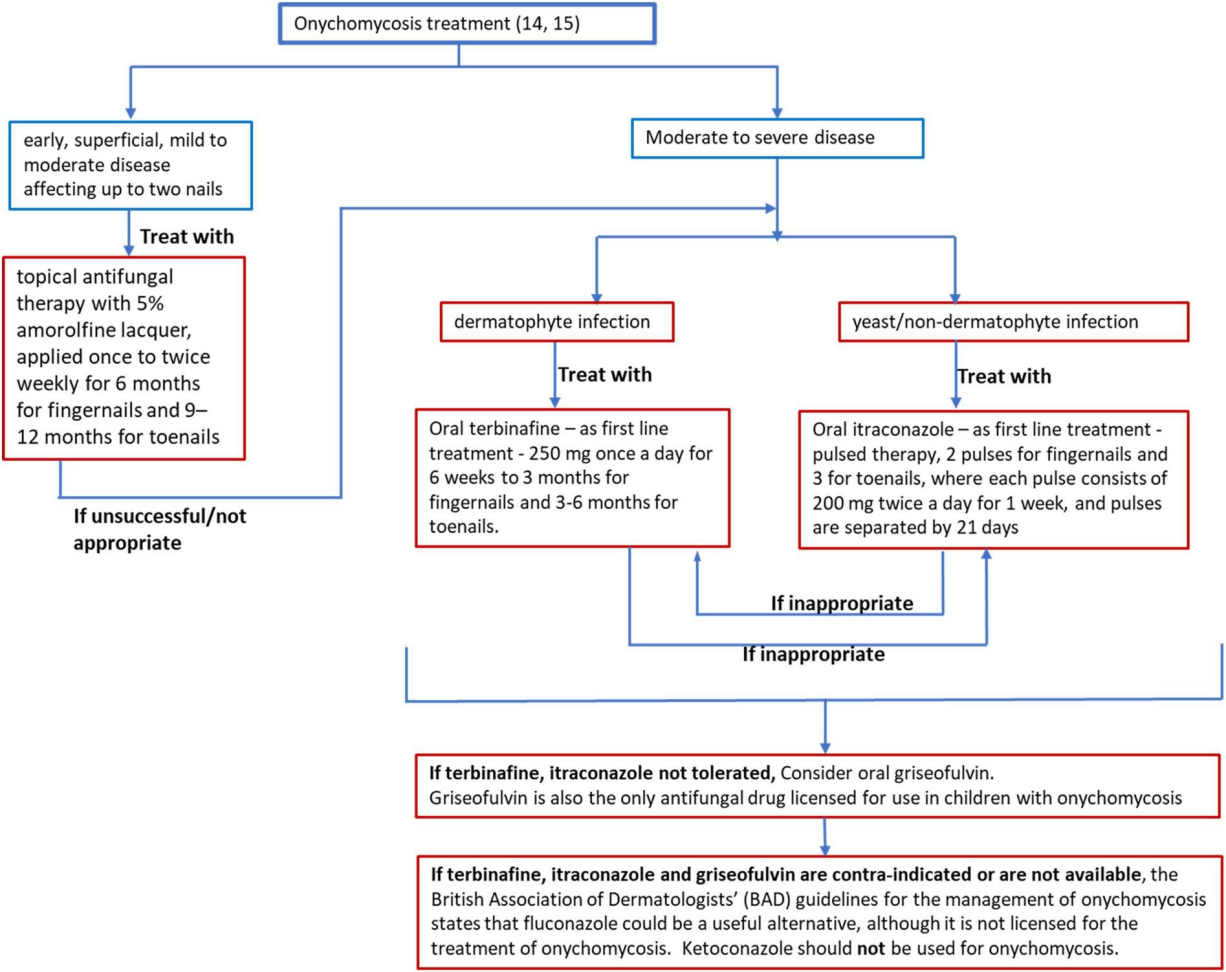
Treatment of onychomycosis as recommended by NICE and BAD^14^, ^15^

In the UK, the National Institute for Clinical Excellence (NICE) recommends topical antifungal therapy with 5% amorolfine lacquer, oral terbinafine, oral itraconazole, oral griseofulvin as per Figure 1.^15^ Griseofulvin is the only antifungal drug licenced for use in children with onychomycosis. If terbinafine, itraconazole and griseofulvin are contra‐indicated or are not available, the British Association of Dermatologists' (BAD) guidelines for the management of onychomycosis^16^ states that fluconazole could be a useful alternative, although it is not licenced for the treatment of onychomycosis. Ketoconazole should not be used for onychomycosis^16^ (Figure 1).

In addition to the NICE and BAD guidelines referred to above, there is an abundance of literature, including from Public Health England (PHE), systematic reviews, meta‐analyses and expert opinions on the optimal clinical management of onychomycosis. In contrast to the slew of publications on how best to treat onychomycosis, information on how onychomycosis is actually treated by general practitioners (GPs) in primary care in the UK is scarce. Information on actual practice is important as it can reveal a host of things, such as to what extent national guidelines are followed and which population groups seek treatment.

The aim of the study was therefore to describe the pattern of onychomycosis treatment in primary care in the UK, stratifying by patient gender and age.

## 3 METHODS

To quantify UK national data for the estimation of onychomycosis incidence, The Health Improvement Network (THIN) database was used. We accessed the data from the IQVIA Medical Research Data (IMRD) that incorporates data supplied by THIN, a propriety database of Cegedim SA. THIN data is unobtrusive medical data collected from over 550 General Practitioners' (GP) primary care records within the UK.^17^ THIN data has been collected since 1994 and comprises information for approximately 6% of patients within the UK, equating to an estimated 3.7 million active patients. It is compliant with all general data protection regulation (GDPR) laws, thus, researchers can extract anonymised patient medical data through the use of software such as the Statistical Package for the Social Sciences (SPSS). All recorded clinical data within THIN is coded. Read codes, also known as medcodes, are a standardised system of clinical terminology for medical conditions, while drug codes are codes for prescribed medications. Read codes and drug codes are used by healthcare professionals to describe the diagnosis, care and treatment given to patients in the community. These validated codes were used to extract data from the THIN database. The study protocol was approved by the THIN Scientific Review Committee (Reference Number: 19THIN073).

This research is a cross‐sectional study reviewing data from GPs on the treatment of onychomycosis. The study population consisted of all patients registered with general practice surgeries taking part in the THIN database with a diagnosis of onychomycosis from 2001 until 2017. A positive onychomycosis diagnosis was classified as patients having the diagnostic Read code for onychomycosis. Incidence rate (number of new cases/population in the THIN database) of onychomycosis in males and females was calculated annually. The use of oral and topical anti‐fungal medicines was reviewed to understand the management of onychomycosis. The anti‐fungal drugs investigated include: terbinafine, itraconazole, griseofulvin, ketoconazole, fluconazole, amorolfine and tioconazole.

### 3.1 Statistical analysis

Statistical software of SPSS (version 17) was used to analyse the data. Frequency tables were created for drug‐use analysis categorising by gender and age. Pearson's Chi‐squared test was used to compare between the categorical variables. A two‐sided *p*‐value of less than 0.05 was used to identify statistically significant results, and confidence interval was set at the 95% level.

## 4 RESULTS

### 4.1 Patient demographics

From the total THIN database population in the years 2001–2017, 42,653 patients had had a diagnosis of onychomycosis, that is, an incidence rate of about 50 per 100,000. The youngest patient diagnosed with and treated for onychomycosis was under 10 years old while the oldest was 90 years or older, with people aged 50–59 years representing the largest group which had sought treatment (Table 1). Gender also had an influence; more males than females had sought treatment for onychomycosis from their GPs, comprising 52% of the cohort, with females making up 48% (Table 2).

**TABLE 1:** Numbers of patients with a diagnosis of onychomycosis, and patterns of antifungal prescribing, stratified by age group. Patient cohort *N* = 42,653

Patient cohort *N* = 42,653 (%)											
Age group	Under 10	10–19	20–29	30–39	40–49	50–59	60–69	70–79	80–89	90 and older	*p*‐value
*N* (%)	539 (1.3)	1803 (4.2)	4229 (9.9)	6328 (14.8)	7404 (17.4)	7881(18.5)	7522 (17.6)	4827 (11.3)	1912 (4.5)	208 (0.5)	
Using oral drugs											
Terbinafine	87 (16.1)	722 (40.0)	2051 (48.5)	3265 (51.6)	4093 (55.3)	4187 (53.1)	3798 (50.5)	2212 (45.8)	736 (38.4)	65 (31.2)	<0.001
Itraconazole	34 (6.3)	181 (10.0)	471 (11.1)	789 (12.5)	1018 (13.7)	1012 (12.8)	806 (10.7)	462 (9.6)	114 (6.0)	9 (4.3)	<0.001
Griseofulvin	26 (4.8)	36 (2.0)	46 (1.1)	73 (1.2)	95 (1.3)	102 (1.3)	97 (1.3)	54 (1.1)	20 (1.0)	2 (1.0)	<0.001
Ketoconazole	0 (0.0)	3 (0.2)	5 (0.1)	7 (0.1)	16 (0.2)	18 (0.2)	6 (0.1)	15 (0.3)	1 (0.1)	0 (0.0)	0.050
Fluconazole	9 (1.7)	112 (6.2)	405 (9.6)	646 (10.2)	779 (10.5)	810 (10.3)	645 (8.6)	398 (8.2)	144 (7.5)	5 (2.4)	<0.001
Using topical drugs											
Terbinafine	81 (15.0)	223 (12.4)	356 (8.4)	574 (9.1)	713 (9.6)	784 (9.9)	785 (10.4)	541 (11.2)	199 (10.4)	21 (10.1)	<0.001
Amorolfine	206 (38.2)	612 (33.9)	1133 (26.8)	1773 (28.0)	2199 (29.7)	2474 (31.4)	2631 (35.0)	1730 (35.8)	658 (34.4)	63 (30.3)	<0.001
Griseofulvin	0 (0.0)	1 (0.1)	1 (0.0)	0 (0.0)	2 (0.0)	4 (0.1)	1 (0.0)	2 (0.0)	1 (0.1)	0 (0.0)	0.814
Ketoconazole	10 (1.9)	19 (1.1)	46 (1.1)	80 (1.3)	92 (1.2)	107 (1.4)	100 (1.3)	63 (1.3)	18 (0.9)	2 (1.0)	0.753
Clotrimazole (with oral fluconazole)	0 (0.0)	14 (0.8)	25 (0.6)	42 (0.7)	44 (0.6)	36 (0.5)	18 (0.2)	8 (0.2)	2 (0.1)	2 (1.0)	<0.001
Tioconazole	101 (18.7)	184 (10.2)	289 (6.8)	432 (6.8)	596 (8.0)	647 (8.2)	653 (8.7)	435 (9.0)	146 (7.6)	18 (8.7)	<0.001

**TABLE 2:** Numbers of patients with a diagnosis of onychomycosis, and patterns of antifungal prescribing, stratified by gender. Patient cohort *N* = 42,653

Numbers (%)			
Total	Male 22,173 (52)	Female 20,480 (48)	*p*‐value
Antifungal medications			
Oral			
Terbinafine	12,112 (54.6)	9104 (44.5)	<0.001
Itraconazole	2484 (11.2)	2412 (11.8)	0.063
Griseofulvin	319 (1.4)	232 (1.1)	0.005
Ketoconazole	29 (0.1)	42 (0.2)	0.060
Fluconazole	963 (4.3)	2990 (14.6)	<0.001
Topical			
Terbinafine	2318 (10.5)	1959 (9.6)	0.002
Amorolfine	6406 (28.9)	7073 (34.5)	<0.001
Griseofulvin	9 (0.0)	3 (0.0)	0.110
Ketoconazole	297 (1.3)	240 (1.2)	0.121
Clotrimazole cream and fluconazole capsule	9 (0.0)	182 (0.9)	<0.001
Tioconazole	1672 (7.5)	1829 (8.9)	<0.001

### 4.2 Drug treatment

The oral and topical drugs prescribed to patients diagnosed with onychomycosis are shown in Tables 1 and 2.

Oral terbinafine was the most commonly prescribed treatment, with 50% of the patients receiving it, followed by topical amorolfine, which was prescribed to 32% of patients. Oral itraconzole, topical terbinafine and topical tioconazole were each prescribed to approximately 10% of patients while a much smaller % of patients received oral griseofulvin (1%), oral ketoconazole (0.2%), topical griseofulvin (0.5%), topical ketoconazole (1%). Fluconazole was also prescribed, but much more commonly to women, and is discussed under the influence of gender.

For oral administration, terbinafine, itraconazole, griseofulvin and ketoconazole were prescribed as tablets, capsules, solutions and suspensions. For topical administration, amorolfine, tioconazole, terbinafine, ketoconazole and griseofulvin were prescribed in the form of nail lacquer, cream, gel, solution, spray or shampoo. The latter was obviously not prescribed for the treatment of onychomycosis, but is included here to show that some patients with onychomycosis also suffered from fungal infections of the scalp.

### 4.3 Influence of patient age

Drug treatment by patient age is shown in Table 1. A clear influence of age can be seen. Compared to the other age groups, the under‐10 year olds had the largest proportion of patients to receive oral griseofulvin (5%), topical amorolfine (38%), topical tioconazole (19%) and topical terbinafine (15%) and the smallest percentage of patients to receive oral terbinafine (16%) and oral fluconazole (2%). At the other end of the age range, the over‐79 year olds had the smallest proportion of patients to receive oral itraconazole (6%). Oral ketoconazole was not prescribed to the under‐10s or to those aged 90 and over.

### 4.4 Influence of patient gender

While oral terbinafine was the most commonly used drug in both males and females, a greater % of men were prescribed it (55% of men vs. 45% of women; *p* < 0.001). A greater % of males compared to females being prescribed terbinafine also held true when fluconazole was removed from the calculations (47% vs. 40% instead of 55% vs. 45% when fluconazole was included in calculations). In contrast, a much larger percentage of women were prescribed topical amorolfine compared to men (35% vs. 29%, *p* < 0.001). Similarly, topical tioconazole was prescribed more commonly (*p* < 0.001) to women than to men, who instead received more topical terbinafine and more oral griseofulvin prescriptions (*p* ≤ 0.005). There was no influence of gender on itraconazole prescription, with 11% of males and 12% of females receiving it (*p* > 0.05), or on ketoconazole prescription which was given to very few patients (*p* > 0.05). Oral fluconazole, with or without clotrimazole, was prescribed to a much greater proportion of women compared to men (*p* < 0.001).

## 5 DISCUSSION

This is, to our knowledge, **the first study showing how onychomycosis is actually treated in primary care** (Tables 1 and 2). The overall incidence rate of 50 per 100,000 people is likely to be an under‐estimate of the true incidence rate in the UK, as many people may not have sought treatment from their GPs while many others may have been self‐treating, with over‐the‐counter medicines and advice from pharmacies.

The **higher proportion of males compared to females (52%** vs. **48%) in the population seeking treatment** for onychomycosis from their GP is a little surprising, given that females are known to be more likely to consult healthcare providers.^18^ However, the higher proportion of males seeking treatment for onychomycosis reflects the greater prevalence of onychomycosis in males compared to females in the UK, where the male/female prevalence ratio is reportd to be 1.1.^3^ Similarly, higher onychomycosis prevalence in males have been reported in North America and in Europe, where the male/female prevalence ratios of 1.9 and 1.5 respectively have been found.^5^ Greater onychomycosis prevalence in males is not universal however. For example, a meta‐analysis showed greater prevalence in women (which could possibly be linked to greater treatment‐seeking) in Iran^19^ and no statistical difference in Crete.^20^ While the reasons for the greater onychomycosis prevalence in males in some parts of the world have not been established, more frequent nail injuries, occupational factors, greater use of occlusive footwear and different hormone levels have been postulated as possible contributors, while cultural differences between the sexes regarding footwear, bathing habits, occupation or sporting activity have been proposed for the different male:female prevalence ratios in different countries.^21^

The **lower observed incidence of onychomycosis in children and young adults** in our study also reflects reports of the lower prevalence in children in the UK^3^ and elsewhere, for example in Senegal,^22^, ^23^ and globally,^7^ with the lower prevalence being attributed to faster nail growth, trauma to the nail in children being less common than in adults in hard‐labouring jobs and a difference in the structure of the nail plate which may be less conducive to colonisation by micro‐organisms.^23^

Table 1 also shows that **50–59 year olds comprised the largest group having treatment for onychomycosis**, followed by 60–69 year olds, then 40–49 year olds. This was slightly surprising, given that onychomycosis prevalence increases with increasing age, with approximately 20% of people over 60 years old and 50% of those over 70 years old suffering from the condition,^7^ due to repeated nail trauma, poorer peripheral blood circulation, slower nail growth and the higher prevalence of co‐morbidities such as diabetes, HIV and psoriasis in older populations.^14^, ^19^, ^23^, ^24^, ^25^ From the known prevalence of onychomycosis, one would have expected the 70 and 70+ year old patients to make up the bulk of the cohort receiving treatment. Our results showed that while onychomycosis may be more present in the older age groups in our population, few of them have sought treatment. Possible reasons for not seeking treatment could be that many older people are less bothered by onychomycotic nails or they may be on medication for other conditions which preclude anti‐onychomycotic drugs due to drug interactions or they may not be able to self‐administer topical medicines, for example, if they cannot reach their toenails or they may previously have tried and failed to treat onychomycosis, and given up.

Onychomycosis treatment: **oral terbinafine being the most commonly used drug** in both men and women reflects numerous reports of its higher efficacy compared to those of other oral antifungals against dermatophyte (the main cause of nail fungal) infections, and guidelines by NICE.^15^ The lower percentage of women prescribed terbinafine compared to men could be due to more diagnoses of *Candida* nail infection, which is more common in women than in men, and in which case, terbinafine is not recommended as first‐line oral treatment due to its lower anti‐Candida activity.^15^, ^16^ For *Candida* nail infections, itraconazole is the first‐line oral treatment (Figure 1), which would explain the slightly higher % of women compared to men who were prescribed it.

**Following terbinafine in pole position, was topical amorolfine lacquer**, used by about a third of patients in all age groups, but with **noticeably higher use in women and the younger (under 10) and older (60–89) age groups.** Higher amorolfine use by women could be due to greater diagnoses of Candidal infections (as discussed above). Given that fingernails are more commonly affected by *Candida* than toenails, and that fingernails are more visible and their appearance is therefore more important, fingernail fungal infections could have led to a greater demand for treatment. **Greater use of the topical amorolfine in older age groups** could be because oral treatment was inappropriate, due to drug interactions or contra‐indications, in older adults who are more likely to be suffering from co‐morbidities and be taking several other drugs. **Greater amorolfine use in children** could be due to the fact that the two most effective anti‐onychomycotic drugs, terbinafine and itraconazole, are not licenced for use in children, leaving fewer treatment options and the selection of a topical product (amorolfine nail lacquer or tioconazole nail solution) for a greater proportion of children. Terbinafine and itraconazole were however prescribed to some children, reflecting guidance in the British National Formulary for Children (BNFC). Oral griseofulvin was also prescribed to children, although less so than terbinafine and itraconazole, again reflecting BAD guidelines^16^ and BNFC which advises its use where topical therapy has failed or is inappropriate.

It must be noted that while for some medicines, for example amorolfine nail lacquer, we can assume that the medicine was prescribed for onychomycosis, this is not universal and is exemplified by the high percentage of women prescribed fluconazole. The latter is often used to treat vaginal and vulval candidiasis in women and to treat penile thrush in men. It is likely therefore that the majority of fluconazole use in Table 2 was not used to treat onychomycosis itself. Similarly, it can be said that ketoconazole shampoo was unlikely to have been prescribed for the treatment of onychomycosis.

It must also be noted that while Tables 1 and 2 show the frequencies and percentages for each drug, some patients would have been prescribed more than one drug concomitantly. For example, oral terbinafine in combination with topical amorolfine is sometimes used as the combination is more effective than monotherapy.^11^ Consequently, the percentages add up to more than 100%, even when fluconazole is removed from the calculation (if it is assumed that all fluconazole in Tables 1 and 2 was used to treat conditions other than onychomycosis).

### 5.1 Limitations of this work

Our analysis was focussed on the treatment of onychomycosis by General Practitioners. The THIN database does not include data from secondary healthcare settings or specialist clinics. In addition, many patients are likely to be self‐treating with over‐the‐counter medication from pharmacies. Our calculated incidence rates are therefore an underestimate. It is also important to note that our incidence rates were extracted based on GP Read codes. As these do not specify whether onychomycosis diagnosis is based on clinical observation or mycological tests, there may be variations in diagnostic accuracies, which could be a potential source of error.

From the THIN database, we classified patients with onychomycosis by utilising the Read diagnostic code. Consequently, patients with onychomycosis, but without an official diagnosis were excluded from the analysis, regardless of their use of oral and/or topical antifungals. Our results are therefore likely to be an underestimation of onychomycosis treatment by GPs. Conversely, as mentioned above, we cannot be certain that all the antifungals prescribed to patients with onychomycosis were specifically used to treat onychomycosis, as some of the drugs, particularly oral fluconazole and ketoconazole shampoo, are likely to been used to treat other fungal infections, such as those of the genitals and skin/scalp respectively. It was not possible, in this study, to definitely establish the diagnosis related to each prescription of an antifungal drug. This should be addressed in future work, as well as the temporal relationships between the diagnoses of different fungal infections, which would also help to establish which fungal infection is a source or a consequence of another fungal infection in an individual or whether there is no relationship.

Another limitation is that due to the nature of the study, the outcome of the treatment was not available for the study. Further studies are encouraged to explore the effectiveness of the treatments.

## 6 CONCLUSIONS

This is the first study which shows how onychomycosis is actually treated in primary care in the UK, and the influences of patient age and gender. Overall, the findings were broadly in concordance with national guidelines about how onychomycosis should be treated.

## CONFLICT OF INTEREST

None to declare.

## AUTHOR CONTRIBUTIONS

**M. Sajeed:** Conceptualization; Formal analysis; Investigation Methodology; Writing – original draft. **L. Wei:** Conceptualization; Project administration;Resources; Software; Supervision; Writing – review & editing. **S. Murdan:** Conceptualization; Project administration; Writing – review & editing.

## Data Availability

Author elects not to share data.
